# Predicting coronary artery disease: a comparison between two data mining algorithms

**DOI:** 10.1186/s12889-019-6721-5

**Published:** 2019-04-29

**Authors:** Haleh Ayatollahi, Leila Gholamhosseini, Masoud Salehi

**Affiliations:** 1 Health Management and Economics Research Center, Iran University of Medical Sciences, Tehran, Iran; 2 Department of Health Information Management, School of Health Management and Information Sciences, Iran University of Medical Sciences, Tehran, Iran; 3 School of Paramedical Sciences, AJA University of Medical Sciences, Tehran, Iran; 4 Department of Biostatistics, School of Public Health, Iran University of Medical Sciences, Tehran, Iran

**Keywords:** Coronary artery disease (CAD), Data mining algorithms, Artificial neural network (ANN), Support vector machine (SVM)

## Abstract

**Background:**

Cardiovascular diseases (CADs) are the first leading cause of death across the world. World Health Organization has estimated that morality rate caused by heart diseases will mount to 23 million cases by 2030. Hence, the use of data mining algorithms could be useful in predicting coronary artery diseases. Therefore, the present study aimed to compare the positive predictive value (PPV) of CAD using artificial neural network (ANN) and SVM algorithms and their distinction in terms of predicting CAD in the selected hospitals.

**Methods:**

The present study was conducted by using data mining techniques. The research sample was the medical records of the patients with coronary artery disease who were hospitalized in three hospitals affiliated to AJA University of Medical Sciences between March 2016 and March 2017 (*n* = 1324). The dataset and the predicting variables used in this study was the same for both data mining techniques. Totally, 25 variables affecting CAD were selected and related data were extracted. After normalizing and cleaning the data, they were entered into SPSS (V23.0) and Excel 2013. Then, R 3.3.2 was used for statistical computing.

**Results:**

The SVM model had lower MAPE (112.03), higher Hosmer-Lemeshow test’s result (16.71), and higher sensitivity (92.23). Moreover, variables affecting CAD (74.42) yielded better goodness of fit in SVM model and provided more accurate result than the ANN model. On the other hand, since the area under the receiver operating characteristic (ROC) curve in the SVM algorithm was more than this area in ANN model, it could be concluded that SVM model had higher accuracy than the ANN model.

**Conclusion:**

According to the results, the SVM algorithm presented higher accuracy and better performance than the ANN model and was characterized with higher power and sensitivity. Overall, it provided a better classification for the prediction of CAD. The use of other data mining algorithms are suggested to improve the positive predictive value of the disease prediction.

## Background

Cardiovascular diseases are among the common diseases in both developed and developing countries and regarded as the main cause of death throughout the world [1]. In fact, any condition or disease that affects the heart, its vessels [2], and the blood circulatory system can be related to coronary vascular diseases (CVDs) [3]. In general, the clinical spectrum of CVDs ranges from asymptomatic ischemia to chronic stable angina pectoris, unstable angina (UA), acute myocardial infarction (AMI), ischemic cardiomyopathy and sudden death [4]. They are sometimes associated with conditions such as hypertension, stroke, coronary artery diseases, chronic heart failure, congenital heart disease, rhythm disorders, subclinical atherosclerosis, valvular disease, and peripheral arterial disease [5]. In recent years, in addition to the main risk factors, other factors such as infection, inflammatory and chronic diseases have been discussed as other risk factors of cardiovascular diseases [6].

At the beginning of the twentieth century, 10% of all the deaths were attributed to cardiovascular diseases. At the end of this century, the mortality caused by CVDs increased to 25%. It is estimated that, considering the present increasing trend, over 35–60% of deaths worldwide would be due to cardiovascular diseases by 2025 [7]. Based on the report by WHO, in 2017, more than half (54%) of the deaths around the world were caused by 10 leading causes, and cardiovascular diseases which led to 15 million deaths in 2015 constituted the largest group of fatal diseases [8]. Cardiovascular diseases kill millions of people annually and this value may be increased up to 24.8 million by 2020 if preventive measures are not taken [9].

In Iran, the Ministry of Health reported that 39.9% of the mortality rate in the country is due to cardiovascular diseases and their risk factors, among them CAD is the most prevalent type and is greatly increasing [10]. CAD is a multi-causal disease, in which a series of risk factors, e.g. increased cholesterol, hypertension, diabetes and smoking should be taken into account [11]. According to the results of an epidemiological study with the aim of examining coronary artery disease mortality rate, 63 out of 6537 death cases were due to CAD in 2015 [12]. CAD is more prevalent among men than women, and the symptoms of the disease may appear in women 10 years later than in men [13]. Therefore, considering the great increase in cardiovascular diseases which imposes a heavy financial burden on the society, medical communities attempt to find a way for the accurate and timely prediction of CAD by using new statistical techniques, such as data mining [14]. It is noteworthy that the healthcare domain is filled with data. However, the data required for effective decision-making and discovery of hidden patterns are not extracted. By extracting useful data and discovering knowledge from the large volume of medical data, the causes of incidence, growth or the spread of diseases can be identified and physicians can be equipped with valuable information for better decision making. Therefore, many healthcare centers are seeking practical solutions for knowledge discovery by means of data mining techniques [15]. These techniques can help to recognize the patterns and factors influencing diseases [16].

The novel science of data mining is among the 10 developing sciences which have made the next decade face enormous technological evolutions. Using specialized knowledge, it will have extensive applications in the domain of medicine. [17, 18]

The literature review showed that different algorithms such as clustering, classifications, regression and association rules, decision trees, Bayesian network, neural network, multi-layer perceptron with error back propagation algorithm, scaled conjugate gradient (SCG) and support vector machine (SVM) have been used for predicting CAD [19–31]. However, the comparison between the algorithms has not received adequate attention. Among these algorithms, artificial neural network has some advantages, such as high speed, simplicity and capability of solving complex relationships between variables and extracting the non-linear relationships by means of training data. Another algorithm is support vector machine which is the most common and effective machine learning algorithm. SVMs have a powerful theoretical background that used in different activities, such as classification, recognition and prediction in supervised learning [32, 33]. Therefore, the present study aimed to compare the PPV of CAD using artificial neural network (ANN) and SVM algorithms and their distinction in terms of predicting CAD in the selected hospitals.

## Methods

### Study design and setting

The present research was conducted using data mining techniques. The research setting was three selected hospitals affiliated to AJA University of Medical Sciences.

### Participants and sampling

In this study, only medical records of patients with coronary artery disease who were hospitalized in three teaching hospitals between March 2016 and March 2017 were used (*n* = 1324). Other diseases, such as arrhythmia, angina pectoris, acute myocardial infarction, chronic rheumatic heart diseases, congenital heart disease, dilated cardiomyopathy, heart failure, hypertrophic cardiomyopathy, hypertensive heart diseases, ischemic heart diseases, myocardial infarction, mitral regurgitation, mitral valve prolapse, pulmonary stenosis, and pulmonary heart disease were excluded. A unique dataset including the same CAD predicting variables was used for both SVM and ANN techniques.

### Instruments

The data collection instrument was a checklist designed based on the variables used in the guideline of the Cleveland heart disease dataset policy in UCI (University of California) repository. [34] The checklist included 25 variables for predicting CAD. These variables were gender, age, weight, marital status, occupation, address, family history, smoking, comorbidity, diabetes, pulse rate, T.S.T waves, high blood pressure (HBP), cholesterol, triglyceride (TG), hemoglobin (Hgb), blood glucose level, creatinine, systolic blood pressure, diastolic blood pressure, chest pain, low density lipoprotein (LDL), high density lipoprotein (HDL), CAD diagnosis, and the length of hospitalization.

The collected data were controlled by using different methods, such as data preparation, integration, cleaning, normalization and reduction.

### Statistical analysis

After normalization, processing and cleansing, data were entered into SPSS (V23.0) and Microsoft Excel 2013. Moreover, R 3.3.2 was used for statistical computing. The dataset was divided into training and testing sets and to do so, the standard randomized allocation method was used. Consequently, 70% of the records was used for training and 30% was used for testing the models.

### Ethical consideration

The study protocol was approved by the Ethical Clearance Committee of AJA University of Medical Sciences. The data were used anonymously and were kept confidential.

## Results

Initially, the research variables were analyzed in each hospital separately. The results showed that the majority of the patients were men (*n* = 829, 62.7%) with the mean age of 54–62 years old. The rest of the patients were women (*n* = 494, 37.3%) with the mean age of 61–64 years old. The weight comparison between the patients showed that there was a significant difference between CAD and mean weight in Hospitals A and B.

### Socio-demographic predictors of CAD

The frequency distribution of CAD and the type of occupation showed that there was a significant difference between having and not having the occupation. In addition, the frequency distribution of CAD and the place of residence suggested that the majority of the patients (*n* = 1082, 98%) resided in cities. Similarly, the frequency distribution of CAD and a family history indicated that there was a significant difference between having and not having a family history of CAD (*n* = 1049, 79.3%) (*p* < 0.001). Moreover, the results showed that 77.3% (*n* = 1024) of patients were non-smokers and there was a significant difference (*p* < 0.001) among the hospitals in terms of smoking and CAD.

### The predicting variables

**Fig. 1:**
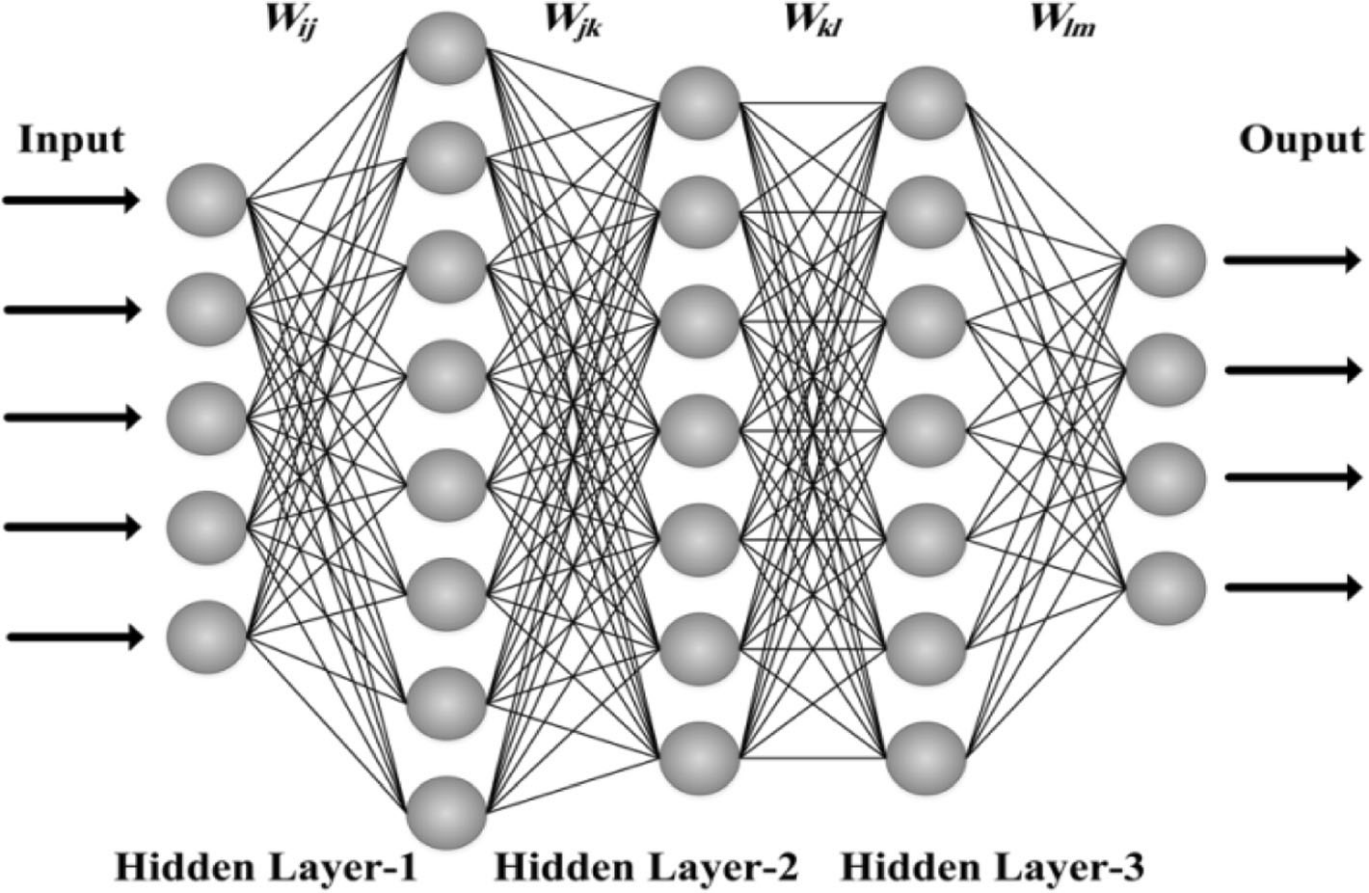
Multi-Layer Perceptron in ANN

The main objective of this study was to determine the PPV of CAD using ANN algorithm and compare the results with the results of the SVM model. Therefore, 25 predicting variables were extracted from the database of cardiovascular patients in the selected hospitals and were used as the input variables and the weight of each was calculated by running algorithms in order to fit the multi-layer ANN model (Fig.1). Based on the calculated weights, the following variables were selected as CAD predicting variables: gender, occupation, place of residence, family history, smoking status, comorbidity, mean value of pulse rate, TST waves status, hypertension history, chest pain, cholesterol, triglyceride, blood glucose level and creatinine level.

In the present study, 70% of the data was used for training and 30% was used for testing the ANN model. The results revealed that the goodness of fit was appropriate in ANN model with the PPV (Available from: https://en.wikipedia.org/wiki/Positive_and_negative_predictive_values) of 0.798, the smaller mean squared error (MSE) and relative error in the test dataset (Table 1).

**Table 1:** PPV indices in ANN algorithm

	Sample	MSE	Relative error	Positive Predictive Value
Training	70%	5.39	0.002	0.798
Testing	30%	3.84	0.002	

$$ \mathrm{PPV}=\frac{\mathrm{number}\kern0.5em \mathrm{of}\kern0.5em \mathrm{true}\kern0.5em \mathrm{positive}\mathrm{s}}{\mathrm{number}\kern0.5em \mathrm{of}\kern0.5em \mathrm{true}\kern0.5em \mathrm{positive}\mathrm{s}\kern0.5em +\kern0.5em \mathrm{number}\kern0.5em \mathrm{of}\kern0.5em \mathrm{false}\kern0.5em \mathrm{positive}\mathrm{s}}=\frac{\mathrm{number}\kern0.5em \mathrm{of}\kern0.5em \mathrm{true}\kern0.5em \mathrm{positive}\mathrm{s}}{\mathrm{number}\kern0.5em \mathrm{of}\kern0.5em \mathrm{positive}\kern0.5em \mathrm{calls}} $$

**Fig. 2:**
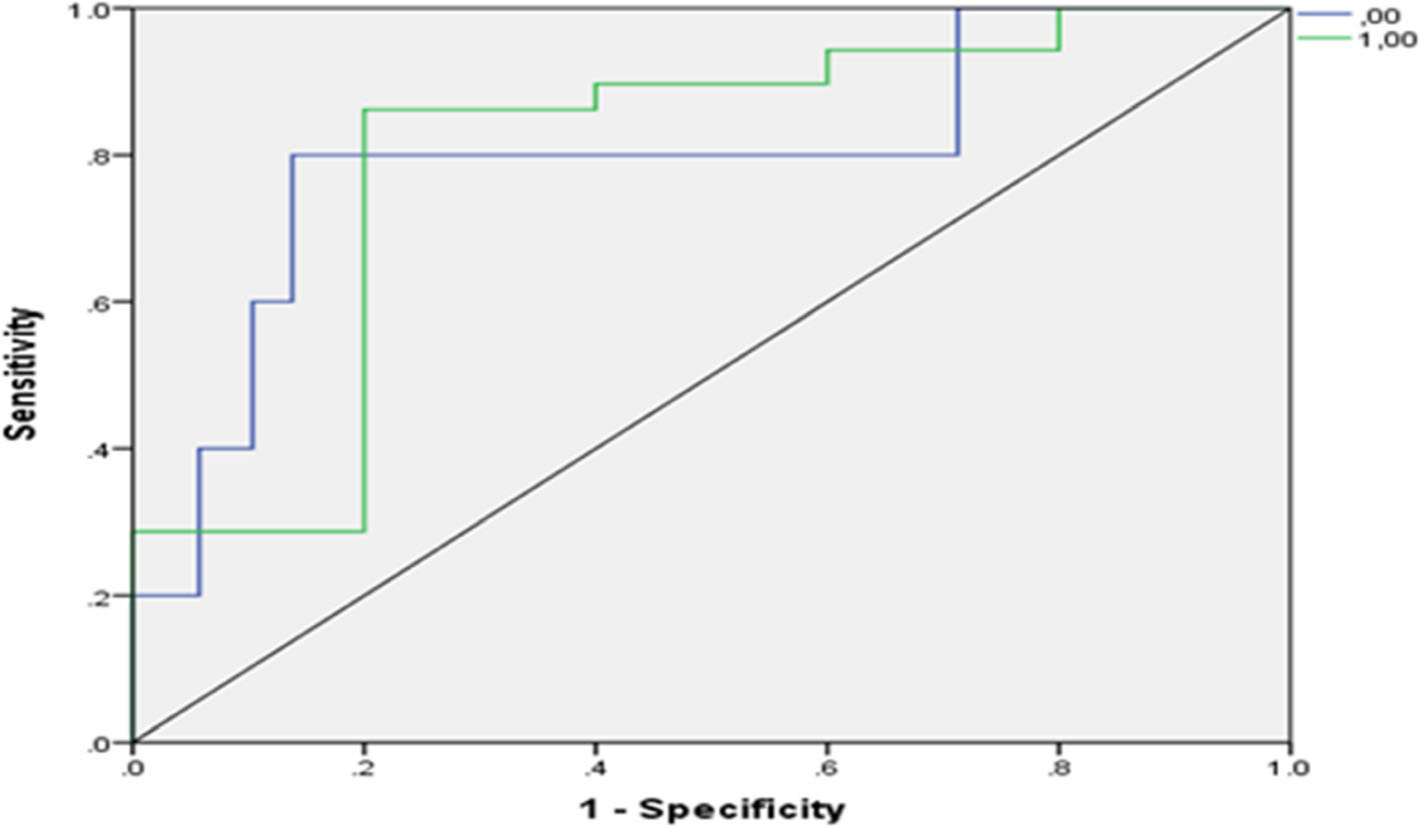
The ROC curve in ANN

Figure 2 illustrates the receiver operating characteristic (ROC) curve for CAD patients. The PPV of the model depends on the extent, to which the test has correctly distinguished CAD patients (sensitivity). This PPV is calculated by computing the area under the ROC curve. The closer this value is to 1, the higher the PPV of the model. Moreover, the closer the value of this ratio is to the left corner, the larger the area under the curve would be. The results showed that the ANN model had high PPV when predicting CAD.

**Fig. 3:**
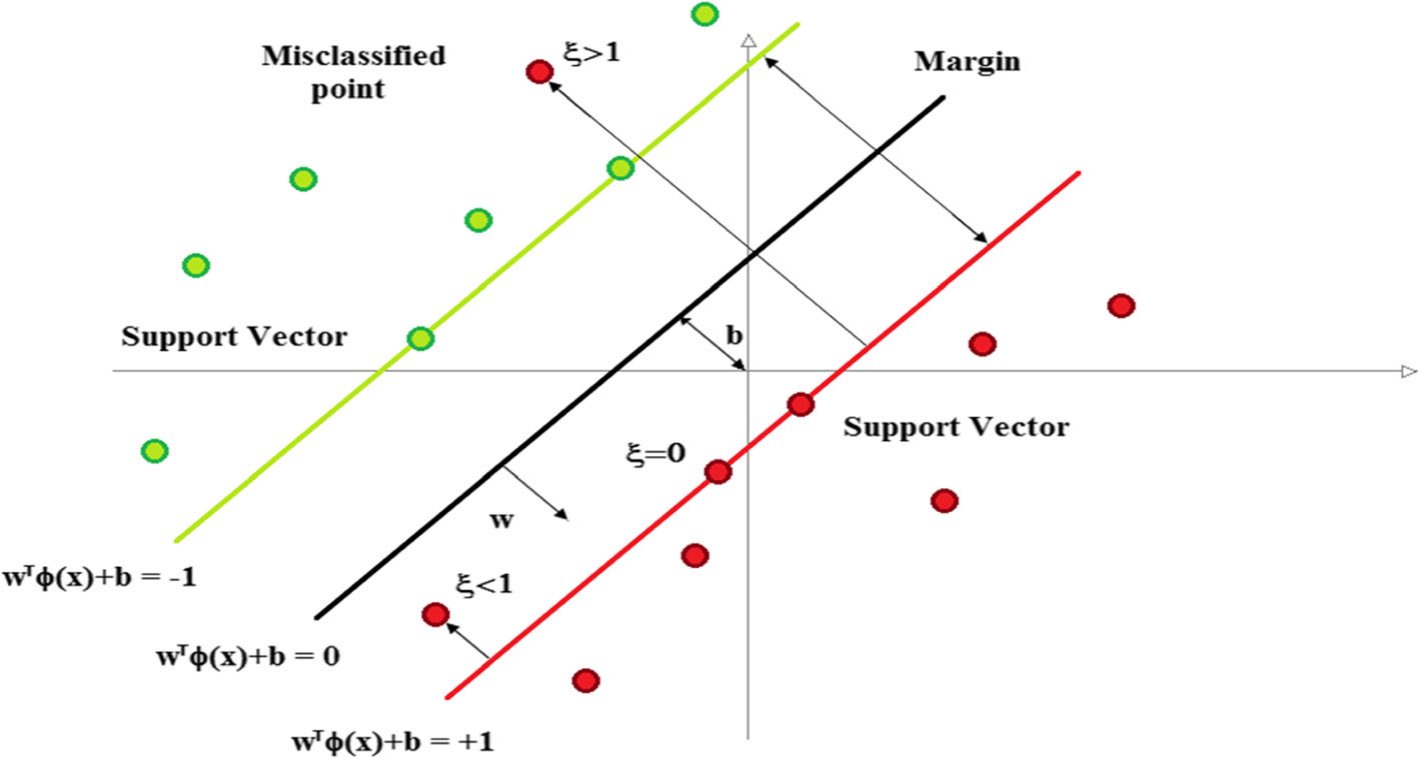
An overview of the SVM model

The PPV was measured by using the SVM algorithm (Fig. 3).

In this phase, 70% of the data were considered as training data and the remaining 30% was used as test data to run the SVM algorithm. Then, PPV of the model is presented in Table 2.

**Table 2:** PPV indices in the SVM algorithm

	Sample	F-measure	Kappa coefficient	Positive Predictive Value
Training	70%	0.761	0.706	0.871
Testing	30%	0.696	0.636

$$ {\mathrm{Cohen}}^{\hbox{'}}\mathrm{s}\ \mathrm{kappa}\ \mathrm{coefficient}=\left(\mathrm{Accuracy}\hbox{-} \mathrm{expAccuracy}\right)/\left(1\hbox{-} \mathrm{expAccuracy}\right) $$

$$ \mathrm{F}\hbox{-} \mathrm{measure}=2\ast \left(\left(\mathrm{PPV}\ast \mathrm{Recall}\right)/\left(\mathrm{PPV}+\mathrm{Recall}\right)\right) $$

As Table 2 shows, F-measure and Cohen’s Kappa coefficients were used to determine the PPV of the SVM model. The result showed that the SVM model had a moderate to high power and sensitivity for predicting CAD patients. Moreover, the SVM model had higher PPV in classifying and predicting CAD. Furthermore, comparison between the accuracy indices showed that, the SVM model had higher accuracy compared to the ANN model and presented better classification (Table 2).

**Fig. 4:**
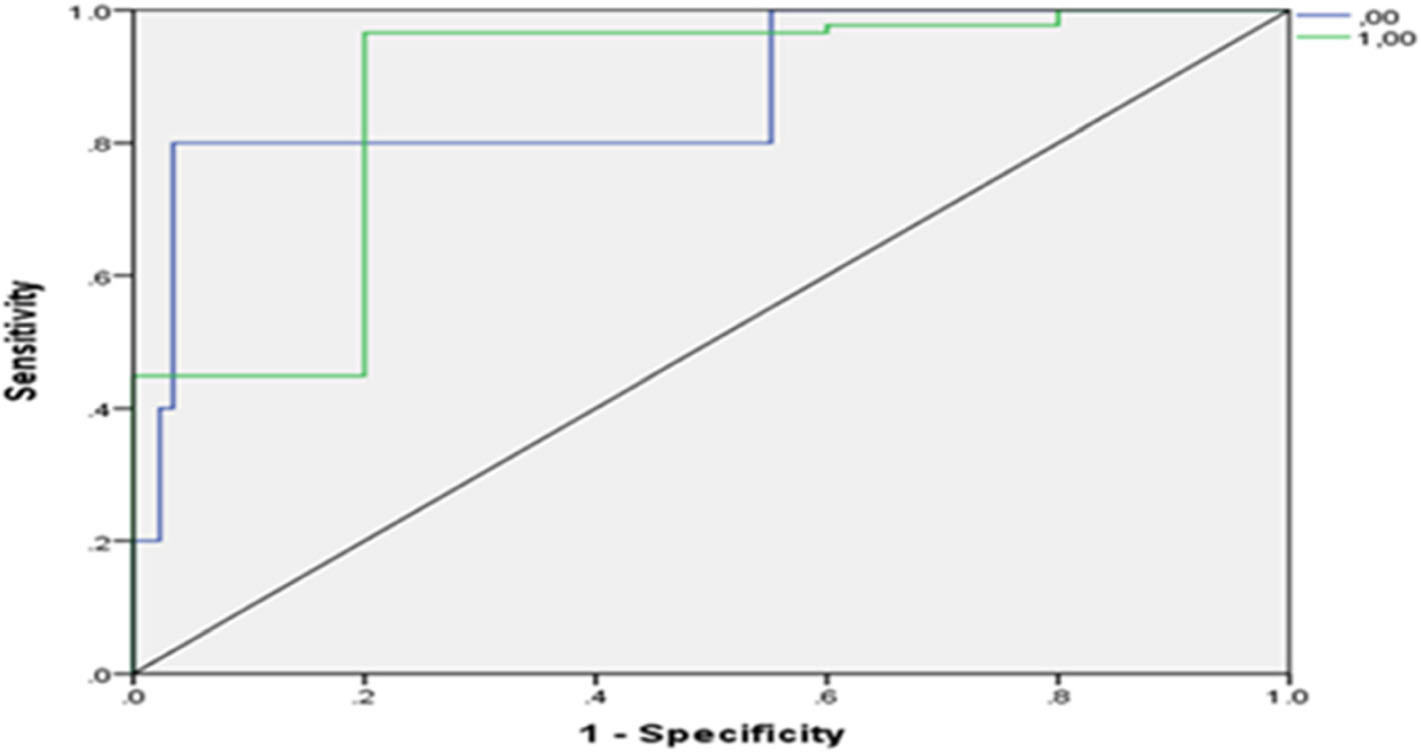
The ROC curve in SVM

That the findings also showed that the area under the ROC curve was larger in the SVM model than in the ANN model (Fig. 4). As a result, SVM had better performance in predicting patients with CAD.

Other statistical tests which were used to compare the performance of the ANN and SVM algorithms were Hosmer-Lemeshow goodness-of-fit test, MAPE (mean absolute percentage error), sensitivity and specificity indices. The tests’ results are presented in Table 3.

**Table 3:** Comparison between the ANN and SVM algorithms

Test	ANN	SVM
MAPE	125.17	112.03
Hosmer-Lemeshow	12.4	16.71
Sensitivity	88.01	92.32
Specificity	73.64	74.42

According to the results, the smaller value of MAPE in the SVM model indicated better fitness of data with less error. Moreover, the larger value of Hosmer-Lemeshow goodness-of-fit test showed the superiority of the SVM model. Furthermore, the SVM model was superior to the ANN model in terms of sensitivity and specificity.

## Discussion

Based on the results, the most important factors affecting the incidence of CAD were gender, occupation, family history, smoking, co-morbidity, mean value of heart rate, TST wave status, hypertension, chest pain, cholesterol, triglyceride, blood glucose level and creatinine. Similarly, previous studies introduced numerous factors affecting the disease and the progress of cardiovascular diseases. These factors were divided into six general groups: environmental factors, daily habits, risk factors, underlying diseases, mental-personality factors and social factors [35]. Other common risk factors associated with CAD include hypertension, lifestyle [36], high level of cholesterol [37], diabetes [38], obesity [39] and smoking [40].

The results of the present study showed that the incidence of the disease was higher in men than women, and the risk of CAD could increase by an increase in age and weight. Similarly, according to another study, age, gender (male) and smoking had significant correlations with CAD [41]. In the study conducted by Masethe and Masethe, a system was proposed for predicting heart attack and included the variables of gender, age, type of chest pain, heart rate, cholesterol, smoking, blood glucose level, blood pressure, diet and alcohol consumption [42].

The findings revealed that the risk of CAD was higher among the employed patients compared to the unemployed and the retired ones. Similarly, the results of a cohort study represented that the risk of cardiovascular diseases was about 40%, because of job strain, and an increase in work load doubled the risk of these diseases. Therefore, the type of job can be a risk factor for cardiovascular diseases [43]. Moreover, the incidence of the disease was higher among those who were living in the urban than the rural areas. In another study, kermani et al. examined the relationship between the mortality rate caused by cardiovascular and chronic obstructive pulmonary diseases (COPD) due to nitrogen dioxide air pollutants in Tehran and reported a significant relationship between these risk factors [44]. Another study investigated the relationship between spatial dispersion of particulate matter and mortality among patients with cardiovascular diseases in Beijing and reported that an increase in particulate matter increased the rate of death among those residing in cities [45]. In another project, researchers evaluated the risk of death by air pollution in 10 cities in Canada and found that there were significant relationships between mortality among patients with cardiovascular respiratory diseases, urban residence and urban air pollutants [46]. However, the results of another study showed that cardiovascular programs have not been implemented in the rural areas; therefore, the mortality rate caused by cardiovascular diseases were increased in the rural areas compared to the big cities [47].

The results also revealed that there was a significant difference between family history and CAD. 193(20.2%) and 215(22.5%) patients had paternal and maternal positive family history (father, mother and siblings) of CAD; there was a possibility to be diagnosed with the disease before 55 and 65 years old in men and women, respectively [48]. As mentioned before, family history of the disease and other risk factors such as blood glucose level, HDL, LDL, cholesterol, systolic and diastolic blood pressure as well as age and gender have been highlighted in the literature [49].

According to the results of the present study, only 58 of the participants were smokers and 142 were non-smokers. The results of a Chi-squared (X^2^) test showed that mean plasma levels of NO was significantly lower in smoker patients (*P* = 0.004). According to the literature, smoking has an increasing trend in Asian countries compared to the rest of the world [50]. Similarly, another study showed that obesity, family history, co-morbidities and smoking can increase the risk of CAD [51]. As smoking is a strong and independent risk factor for cardiovascular diseases, all patients with these diseases must stop smoking [52]. Doctors emphasize that the risk of CAD can be considerably reduced in future by limiting smoking. Therefore, the status of smoking must be systematically evaluated in patients with cardiovascular diseases [39]. According to the results of a hospital-based observational study, there is a direct association between the smoking status and CAD among the young adults. In general, the incidence of CAD had a higher mean value among smokers and the age of patients was lower than or equal to 35 years old [53].

According to the results, 28.6% of the patients had one or multiple co-morbidities. In another study, the results showed that patients with ischemic heart disease (IHD) and chronic obstructive pulmonary disease had the most severe complications compared to those with only one of the noted diseases [54]. Furthermore, according to the results of other studies; obesity, hypertension, diabetes mellitus, metabolic syndrome, high levels of LDL, low level of HDL, high fat diet, lack of regular exercise and dyslipidemia are the risk factors for the mentioned diseases [55, 56].

In terms of the relationship between the mean value of heart rate and the incidence of CAD, a significant relationship was seen which showed, the risk of CAD increases by increasing the mean value of heart rate. The findings of the present study indicated that only 45% of patients had abnormal TST waves and there was no relationship between TST waves’ status and CAD. In a research on the diagnosis of ventricular cardiomyopathy using ANN algorithms, the results showed that a reduction in the dimensions of cardiac signals had a positive effect on the cardiac sound classification [57].

Another finding of the current study was related to the relationship between the incidence of CAD and level of triglyceride and creatinine. In fact, the risk of CAD could increase due to an increase in these variables. Moreover, a significant relationship was seen between the chest pain and CAD [39]. However, the results of the study conducted by wertli et al. showed that there was no significant relationship between these variables. The chest pain has a subjective nature which cannot be used for predicting CAD and panic disorders should be considered in recognizing types of chest pain. [58].

According to the literature review, numerous studies have been conducted to predict CAD by using data mining algorithms. For instance, Kurt et al. used logistic regression, decision trees, classification and neural networks and finally, the multi-layer perceptron ANN with the PPV of 78.8% was introduced as the best model [59]. In another study, Sajja employed a simple Bayesian algorithm, decision tree and multi-layer perceptron ANN on a dataset. The results showed that the precision of the multi-layer perceptron ANN algorithm was 91.75, indicating the best performance [60]. In the present study, CAD was selected as the output variable and 25 variables were used as input variables. The results showed that the ANN model could be appropriate for fitting these data with the total PPV of 0.798. On the other hand, the SVM algorithm fitted the data with smaller MAPE and error. The larger value of Hosmer-Lemeshow goodness-of-fit test also showed the superior performance of the SVM model on the data and provided better prediction for CAD diagnosis. Furthermore, the SVM algorithm predicted CAD patients with higher PPV and sensitivity than the ANN model.

Similarly, the results of the previous studies showed that the use of the SVM algorithm predicts the disease and distinguishes patients from non-patients with higher accuracy [61–63]. Other studies have also confirmed the superior performance and precision of SVM. Nevertheless, there are few studies which do not confirm the efficiency of this algorithm and suggest other methods, such as binary particle swarm optimization (BPSO) and genetic algorithm as the best model of choice for CAD determination [64]. Although input variables were selected based on the literature review and related guidelines, there might be other risk factors which can be studied in the future to gain a bigger picture of the disease risk factors. Moreover, in this study, the results of two algorithms were compared. The data can be used to test other algorithms, such as genetic algorithm to recognize the best performance model.

## Conclusion

The process of disease prediction in medical sciences is as an important process for decision-making and physicians need to know the risk factors for different diseases. This process can be facilitated by using logical and purposeful methods, such as machine learning methods and data mining algorithms. Currently, due to the considerable increase in cardiovascular diseases and the heavy financial burden imposed by them on the society, healthcare communities are seeking ways to predict, diagnose, and treat these diseases effectively. The results of the current study showed that the use of data mining algorithms, such as the SVM model can be useful in predicting CAD. However, more research is needed to compare the performance of different algorithms and to find the best performance model.

## Abbreviations

AMI: Acute Myocardial Infarction
ANN: Artificial Neural Network
BPSO: Binary Particle Swarm Optimization
CAD: Coronary Artery Disease
COPD: Chronic Obstructive Pulmonary Disease
IHD: Ischemic Heart Disease
MAPE: Mean Absolute Percentage Error
MSE: Mean Squared Error
SCG: Scaled Conjugate Gradient
SVM: Support Vector Machine
UA: Unstable Angina
